# Tailoring the Effects of Titanium Dioxide (TiO_2_) and Polyvinyl Alcohol (PVA) in the Separation and Antifouling Performance of Thin-Film Composite Polyvinylidene Fluoride (PVDF) Membrane

**DOI:** 10.3390/membranes11040241

**Published:** 2021-03-28

**Authors:** Shruti Sakarkar, Shobha Muthukumaran, Veeriah Jegatheesan

**Affiliations:** 1 School of Engineering, RMIT University, Melbourne, VIC 3000, Australia; jega.jegatheesan@rmit.edu.au; 2 College of Engineering and Science, Victoria University, Melbourne, VIC 8001, Australia; shobha.muthukumaran@vu.edu.au

**Keywords:** dip-coating, dyes, membrane, nanocomposite, polyvinylidene fluoride, titanium dioxide

## Abstract

In this study, thin-film composite (TFC) polyvinylidene fluoride (PVDF) membranes were synthesized by coating with titanium dioxide (TiO_2_)/polyvinyl alcohol (PVA) solution by a dip coating method and cross-linked with glutaraldehyde. Glutaraldehyde (GA) acted as a cross-linking agent to improve the thermal and chemical stability of the thin film coating. The incorporation of TiO_2_ in the film enhanced the hydrophilicity of the membrane and the rejection of dyes during filtration. The layer of TiO_2_ nanoparticles on the PVDF membranes have mitigated the fouling effects compared to the plain PVDF membrane. The photocatalytic performance was studied at different TiO_2_ loading for the photodegradation of dyes (reactive blue (RB) and methyl orange (MO)). The results indicated that the thin film coating of TiO_2_/PVA enhanced photocatalytic performance and showed good reusability under UV irradiation. This study showed that nearly 78% MO and 47% RB were removed using the TFC membrane. This work provides a new vision in the fabrication of TFC polymeric membranes as an efficient wastewater treatment tool.

## 1. Introduction

Rapid urbanization and industrialization are leading to water scarcity and deterioration of the quality of freshwater. Effluents discharged from industries contain persistent organic pollutants (POP), which are synthetic chemicals with pronounced persistence against chemical or biological degradation and bioaccumulation that have substantial impacts on human health and the environment, even at very minimal concentrations over a prolonged period [1].

Synthetic textile dyes are emerging POPs from the textile industries [2]. The textile industry is the highest water consuming industry and generates a large volume of effluent during the dyeing and finishing processes, where most of the dyes were washed out with water [3]. Effluents generated by the textile mills are notorious for their complexity, comprising synthetic dyes, cleansing agents, salts, surfactants, dispersants, inhibitory compounds, oil, toxic chemicals, and many other compounds [4,5]. The chemicals present in synthetic dyes can cause fatal and mutagenic effects on living beings and aquatic life [6]. Therefore, the main challenge is to reduce and remediate contaminated textile effluents economically and in a sustainable manner. Traditional technologies used to treat textile effluents have limitations and are still not adequate for complete degradation and removal of dye residues, as most of the compounds are highly resilient for these processes to be effective [7]. Among different wastewater treatments, the membrane filtration process has attracted significant attention for textile effluents’ treatment because of its high separation efficiency, simplistic operation, no sludge production, and easy to scale-up. Despite the advantages mentioned above, fouling is the major factor that restricts the application of polymeric membranes for textile effluent treatment [8]. The incorporation of nanoparticles into membranes is a novel approach to overcome the disadvantages of fouling in polymeric membranes.

Thin-film composite (TFC) membrane with nanoparticle incorporated on the superficial layer improves the physico-chemical, mechanical, and thermal properties of the membrane. The advantage of the superficial layer is that it acts as a selective barrier during the separation process, which enhances the separation performance of the membrane. Various types of nanoparticles have been incorporated into the top layer of TFC membranes, such as carbon nanotubes [9], graphene oxide [10], silica [11], zeolites [12], and titanium dioxide [13,14,15,16]. Additional selective transport channels can be formed in TFC membranes due to that porous nanofiller or the interfaces between nanofiller and polymer matrices, facilitating the separation property of the resultant membrane.

Previous studies have shown that TFC membranes have great potential in separating and reducing pollutants from wastewater [17,18]. The incorporation of nanoparticles in the thin film enhances the water permeability, antibacterial, and antifouling properties of the membrane [19].

Titanium dioxide (TiO_2_) is probably the most widely used nanomaterial in membrane modification [20,21] due to their ability to detoxify harmful organic pollutants through their high photocatalytic activity, larger surface area, flexibility in the surface function, and their mechanical stability towards the UV irradiation [22]. Various methods used to immobilize the TiO_2_ nanoparticles for the surface modification of a membrane are dip or spin coating, blending, hot pressing, and physical and chemical cross-linking [23,24].

Cross-linking of TiO_2_ nanoparticles using polymers, such as polyvinyl alcohol (PVA) onto a membrane have been found to have significant application in wastewater treatment [25,26,27]. The idea behind PVA application is to introduce a hydrophilic group into the polymeric membrane to reduce the membrane’s fouling [28]. The oleophobic behavior of PVA decreases the fouling rate and enhances the membrane’s thermal and mechanical properties. The incorporation of TiO_2_ improves photodegradation performance due to the election-hole separation when irradiated by UV light with an energy equal to or greater than the bandgap energy of the TiO_2_ nanoparticles [29]. In our previous work, polyvinylidene fluoride (PVDF) membranes were synthesized by optimizing the PVA content at a fixed TiO_2_ concentration. The optimization was carried out with respect to the water flux and removal of dyes achieved by the membranes [30]. The study showed that 3 wt.% of PVA was the optimum loading for the TFC of the membrane. Li et al. [31] observed synthesized PVDF and PVA hollow fiber membrane modified with TiO_2_ nanoparticles enhanced the dye rejection ability of the membrane compared to the PVA thin film membrane. Liu et al. [32] demonstrated that the thin film membrane composed of PVA and TiO_2_ nanoparticles showed improved photoactivity under visible light [30,33]. All these findings demonstrate that the combination of TiO_2_ and PVA coating of TFC membranes improves the physio-chemical properties of the membrane compared to the conventional polymeric membranes.

This study aims to fabricate a TFC membrane with TiO_2_ nanoparticles incorporated into the PVA layer to improve the antifouling and dye rejection performance. The concentration of TiO_2_ is optimized in this study. The surface chemistry, surface morphology and hydrophilicity of the TiO_2_/PVA incorporated membranes were also characterized at different TiO_2_ loading.

Moreover, the separation capability of TFC membrane was studied using the cross-flow filtration method and ex-situ method for photodegradation of methyl orange (MO) and reactive blue (RB) dyes. The effect of different parameters, such as pH, TiO_2_ loading, and zeta potential, were studied to understand the behavior of the TFC membranes. Furthermore, the kinetic study of photodegradation of dyes by TFC membranes conducted at different TiO_2_ loadings is considered one of the novelties of this paper.

In addition, the stability of the thin film coating is an important parameter that decides the separation performance of the membrane. The stability of the PVA/TiO_2_ coating during multiple filtration runs have not been reported so far. Therefore, in this study, we have focused on the stability of the TFC membranes under UV irradiation during multiple runs.

## 2. Materials and Methods

### 2.1. Materials

All the chemicals used for this research were analytical grade and used without any further purification. Pellet of PVDF (MW = 534,000 g/mol) and PVA (99% hydrolyzed) were obtained from Sigma–Aldrich (NSW, Australia). Cross-linking agent glutaraldehyde (25% in H_2_O) was procured from Sigma–Aldrich (NSW, Australia). MO and RB were used for the separation, whereas bovine serum albumin (BSA) was used to prepare the feed solution to study membrane fouling, and all of them were purchased from Sigma–Aldrich (NSW, Australia). The type, molecular weight, and chemical structures of the dyes used as well as the wavelength at which maximum absorption of those dyes occurred are given in Table 1. TiO_2_ (Aeroxide^®^ P25, (Brunauer–Emmett–Teller, BET) surface area = 35–65 m^2^/g, particle size ≈ 21 nm) were acquired from Sigma–Aldrich (NSW, Australia). N-N dimethylacetamide (DMAc), Sulphuric acid (H_2_SO_4_), sodium hydroxide (NaOH), hydrochloric acid (HCl), and isopropanol (IPA) were acquired from Merck chemicals (Melbourne, Australia).

### 2.2. Methods

#### 2.2.1. Fabrication of PVDF Flat Sheet Membranes

A flat sheet PVDF membrane was fabricated using the non-solvent induced phase separation (NIPS) technique. Dried PVDF (16 wt.%) pellets were mixed mechanically with DMAc (84 wt.%) solvent overnight to obtain a homogeneous solution. Then the solution was sonicated at 60 °C for 30 min for complete degassing. The casting solution is then spread uniformly on a glass plate using a thin film applicator (Elecometer 4340, Manchester, England) at a thickness of 200 µm; then the film was immersed in a non-solvent bath containing a mixture of water (70 vol%) and isopropanol (30 vol%) for the precipitation at room temperature for at least 10 min. Further details of the membrane preparation are described elsewhere [34].

#### 2.2.2. Surface Modification of PVDF Membranes Using TiO_2_/PVA Solution

**Figure 1:**
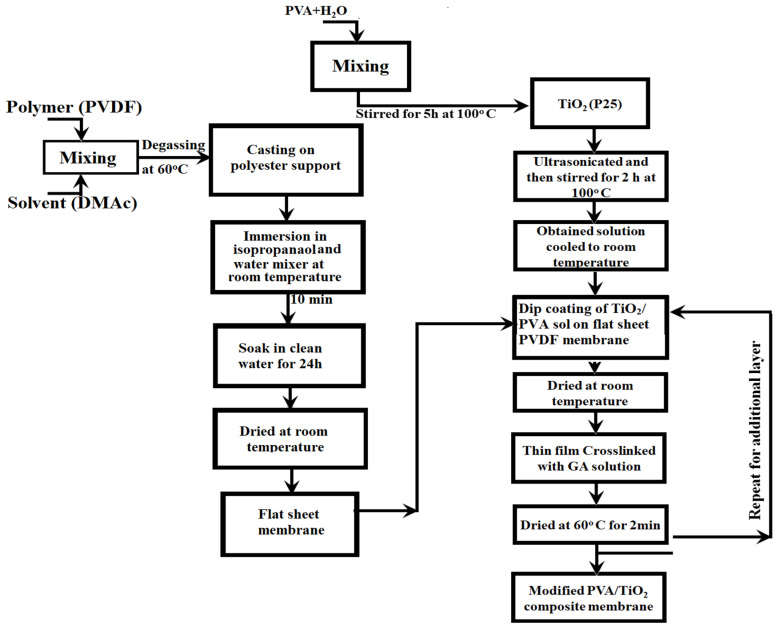
Preparation and synthesis flow diagram for the synthesis of TFC TiO_2_/PVA/ PVDF membrane.

**Figure A1:**
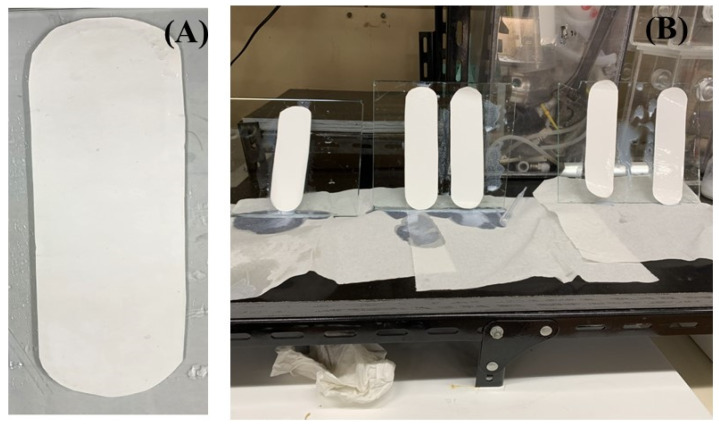
Actual images of the membrane (**A**) neat PVDF membrane (**B**) TiO_2_ /PVA coated PVDF membrane with different TiO_2_ loading (hang them for the removal of excess solution.

TFC PVDF membranes were prepared by chemical cross-linking reaction of PVA and TiO_2_ with GA. PVA was dissolved in water at 100 °C until a transparent solution was obtained, followed by adding of TiO_2_ nanoparticles (Table 2). Viscosities of the solutions were measured by a stress-controlled rheometer (Reologica Merlin II Rheosys). PVDF membrane was washed with a mixture of ethanol and water and spread evenly on a glass plate for complete drying. PVA/TiO_2_ sol was then poured on a clean and dried PVDF membrane and spread evenly all over it. Excess solution was removed by hanging membrane at room temperature (as shown in Appendix A Figure A1). Figure 1 demonstrates the flow diagram for the preparation and synthesis of the TFC PVDF membranes. Lastly, TFC membranes were dried at room temperature before cross-linking.

TFC TiO_2_/PVA/PVDF membrane was soaked in a cross-linking solution consisting of 2 mL GA and 0.5 mL sulfuric acid in water for 5 min at room temperature. The excess solution was removed, and the membrane was dried at 60 °C for 2 min for better cross-linking. Here sulfuric acid acted as a catalyst for the process of cross-linking. The selection of GA and sulfuric acid was based on our previous work recommendations [30]. Each membrane was coated twice to obtained better efficacy for TiO_2_ nanoparticles.

### 2.3. Membrane Characterization

#### 2.3.1. Surface Morphology and Chemical Composition of TiO_2_/PVA Composite PVDF Membrane

The plain and modified TFC PVDF membrane’s surface morphology were examined using a field-emission in- lens scanning electron microscope (FEISEM) (FEI Nova Nano SEM, Hillsboro, OR, USA). Washed and dried membranes were cut into small pieces and sputter-coated with gold particles for electrical conductance. The images were scanned under high voltage at different magnification to understand the presence and dispersion of TiO_2_ nanoparticles over the membrane’s surface. Energy dispersive X-ray (EDX) (Oxford X-Max20 EDX detector) was used to study the membrane’s elemental composition. In this study, EDX was carried out in conjunction with the SEM by using AZtecAM software. Each elemental composition was estimated using the average of three measurements of each sample.

Surface roughness for all the membranes were measured using an atomic force microscope (AFM) (Asylum MFP-3D Infinity). Small pieces (approximately 1.5 cm × 1.5 cm) of the prepared membranes were glued on a flat and hard substrate such as glass. The membrane was scanned and imaged in a scan size of 2 μm × 2 μm. Roughness parameters were obtained and reported in terms of root mean square roughness (RMS) and mean surface roughness (Sa); values are calculated by using Gwyddion software (Version 1.2). Recorded results are the average of three measurements of a sample.

The functional affinity of plain and TFC PVDF membranes were analyzed using Fourier-transform infrared spectroscopy (FTIR) (Thermo FTIR spectrometer, Dreieich, Germany).

The crystalline phases of the membranes with and without thin film coating were compared using high-resolution X-ray diffraction (XRD) (BRUKER-AXS, Karlsruhe, Germany) with the integrated software diffract EVA (Version 09.2017).

The membrane’s hydrophilicity at different TiO_2_ loading in thin-film coating was calculated by the sessile drop method using the OCA20 instrument (Scientex, Melbourne, Australia) at room temperature. Images were captured when droplet fell on the dry membrane surface, and then contact angles with water were measured by supplied software. The reported contact angle is the average of five consecutive measurements of the same membrane at different locations.

#### 2.3.2. Surface Charge of the Membrane

The membrane surface Zeta potential was determined by a streaming potential analyzer (Malvern surface zeta potential). The membrane’s surface charge was measured by adding a small piece (1 mm × 1 mm) of a membrane in milli-Q water and at a required pH. The reported values are the average of three runs of three different samples of each membrane at pH ranging from 3 to 11. The chemicals such as NaOH (base) and HCl (acid) were used to adjust the milli-Q water’s pH.

#### 2.3.3. Filtration Performance and Fouling Analysis

**Figure 2:**
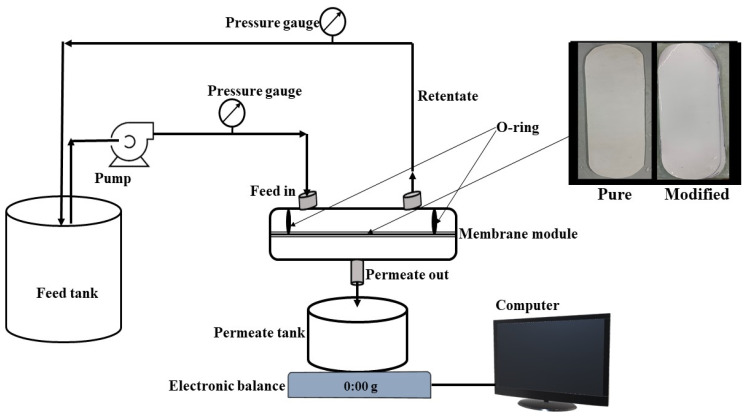
Schematic representation of the cross-flow filtration unit.

The filtration performance of the plain and TFC PVDF membranes’ were analyzed through cross-flow filtration set up (Figure 2). A cross-flow filtration system carried out all the filtration experiments for the membrane with different TiO_2_ loading. The membrane with an active filtration area of 0.00746 m^2^ was placed in the cross-flow filtration unit, and the flow rate was kept constant at 18 L/h at an operating pressure of 3 bar. Milli-Q water was used for the calculation of pure water flux by using Equation (1).

(1) $$J=\frac{V}{A\;\times t}$$

where *V* is the total volume of permeate collected within time *t*, and *A* is the active membrane surface area used for filtration. The two different dyes (RB and MO) were used to prepared model dye solutions at a concentration of 50 mg/L each. The feed solution was circulated throughout the setup for at least 20 min before the reject was collected for the analysis. All filtration experiments were carried out at room temperature.

Samples (feed and permeate) were collected at regular intervals, and change in the concentration of dyes were measured by UV-Vis spectrometer (Shimadzu UV 2700, Kyoto, Japan). The wavelength at which the maximum absorbance of a dye obtained was found for each dye solution to produce calibration curves and to find the concentration of dyes in the samples collected. The rejection percentage, R (%), for each experiment was calculated using Equation (2) given below:

(2) $$R\;\left(\%\right)=\left(1-\frac{C_{p}}{C_{f}}\right)\times 100$$

where C_p_ is the permeate concentration, and C_f_ is the concentration of feed.

#### 2.3.4. Analysis of Membrane Fouling

Fouling effects on plain and TFC PVDF membranes were analyzed as described below:

First, the cross-flow filtration of pure water was carried out for 30 min using the selected membrane. This was followed by the filtration of 1 g/L of BSA solution for another 60 min. The feed BSA solution was recirculated during this period. Then, the cross-flow filtration of pure water was resumed and continued for another 50 min. Permeates of pure water and BSA solution were collected continuously to compute flux changes during this period. The membrane resistance was calculated using the pure water flux obtained before and after the BSA solution’s filtration. The irreversible fouling (IF) factor of the membrane was calculated using Equation (3).

(3) $$\mathrm{IF}=\frac{J_{0}-J_{1}}{J_{0}}\times 100$$

where J_0_ and J_1_ are the pure water flux values before and after the filtration of BSA solution.

#### 2.3.5. Measurement of Photocatalytic Activity

Photocatalytic effectiveness of the TFC PVDF membranes were evaluated by photodegradation of the synthetic dyes (MO and RB) in an aqueous solution. All photocatalytic experiments were studied by the ex-situ method. The prepared nanocomposite membrane (5 cm × 5 cm) was immersed in 130 mL of 50 mg/L of an aqueous solution of a dye and was kept in the dark for 30 min to stabiles the adsorption of dye onto the membrane surface. The solution was then irradiated under two Ultraviolet-C (UV-C) lamps (Philips TUV 15W/G15 T8) with a light intensity of 2.1 mW/cm^2^ each. Samples from the solution were collected at regular intervals to observe the photodegradation of dyes. Change in concentration was examined using UV-spectrometer. Photodegradation (D) was calculated in percentages of dye degradation according to Equation (4):

(4) $$\mathrm{Photodegradation},\;D\left(\%\right)=\frac{C_{i}-C_{f}}{C_{i}}\;\times 100$$

where *C_i_* and *C_f_* are the initial and final concentrations of dye before and after UV irradiation.

## 3. Results and Discussions

### 3.1. The Surface Morphology of TFC Membranes at Different TiO_2_ Loading

**Figure 3:**
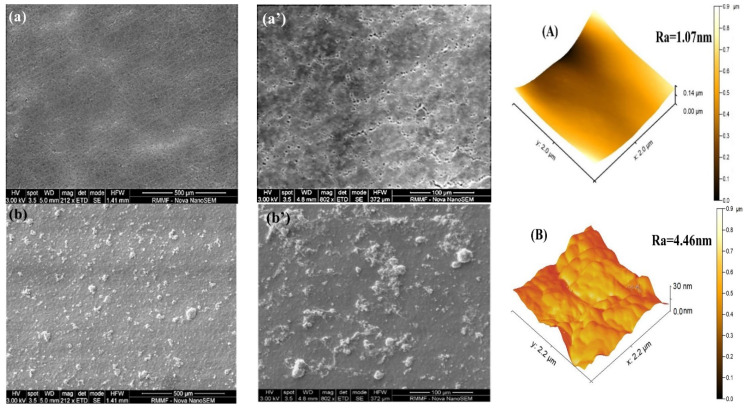

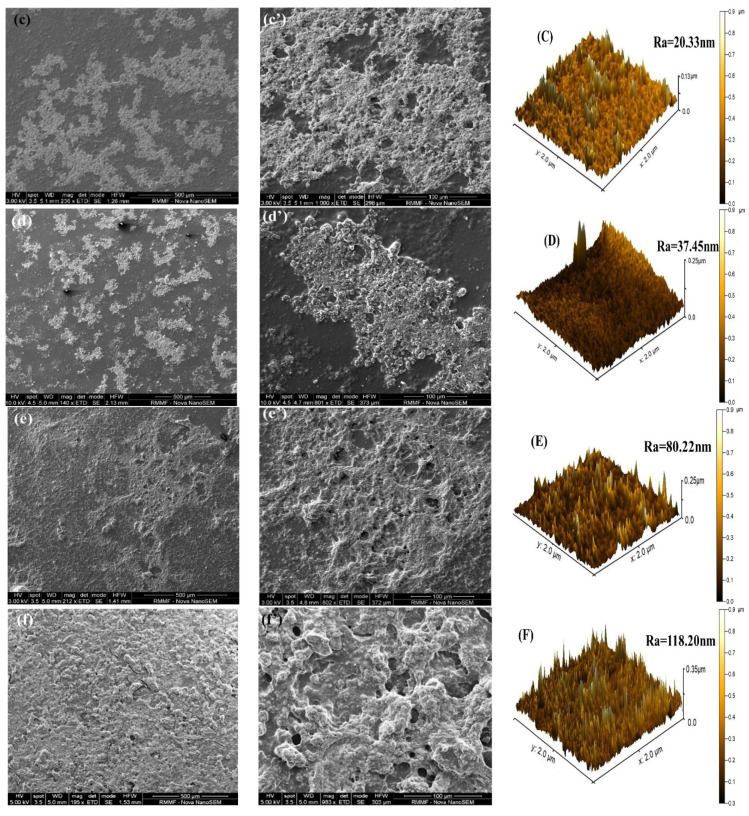
SEM images of the top surface of the TFC membranes at different magnification at different TiO_2_ loading (**a**,**a’**) = 0 wt.% TiO_2_; (**b**,**b’**) = 1 wt.% TiO_2_; (**c**,**c’**) = 1.5 wt.% TiO_2_; (**d**,**d’**) = 2 wt.% TiO_2_; (**e**,**e’**) = 3 wt.% TiO_2_; (**f**,**f’**) = 5 wt.% TiO_2_ and AFM images of the corresponding membranes (**A**) = 0 wt.% TiO_2_; (**B**) = 1 wt.% TiO_2_; (**C**) = 1.5 wt.% TiO_2_; (**D**) = 2 wt.% TiO_2_; (**E**) = 3 wt.% TiO_2_ and (**F**) = 5 wt.% TiO_2._

The surface morphology of the plain and TFC PVDF membranes were investigated using SEM analysis. As illustrated in Figure 3(a,a’), the plain PVDF membrane’s surface has some obvious macro-pores, whereas the modified membranes showed a layer of TiO_2_/PVA over the substrate. The distribution of TiO_2_ nanoparticles was non-uniform at smaller concentrations (i.e., at 1, 1.5, and 2 wt.% TiO_2_); in contrast, the further increase in TiO_2_ loading has led to a uniform distribution over the membrane surface and part of that agglomerated on the surface which can be clearly seen in the SEM images (Figure 3). When the TiO_2_ loading was increased beyond 2 wt.%, the nanoparticles might have incorporated into the membrane’s pores, which will reduce the membrane’s porosity. Porosities of the membranes have been studied in one of our previous works for the membrane containing PVA/TiO_2_ thin film using gravimetric analysis [30]. The aggregation or stacking of nanoparticles may induce a noticeable change in the photocatalytic performance and the stability of the composite membranes. The aggregation of nanoparticles increases the thickness of the TiO_2_/PVA thin film, which decreases the permeate flux.

**Figure 4:**
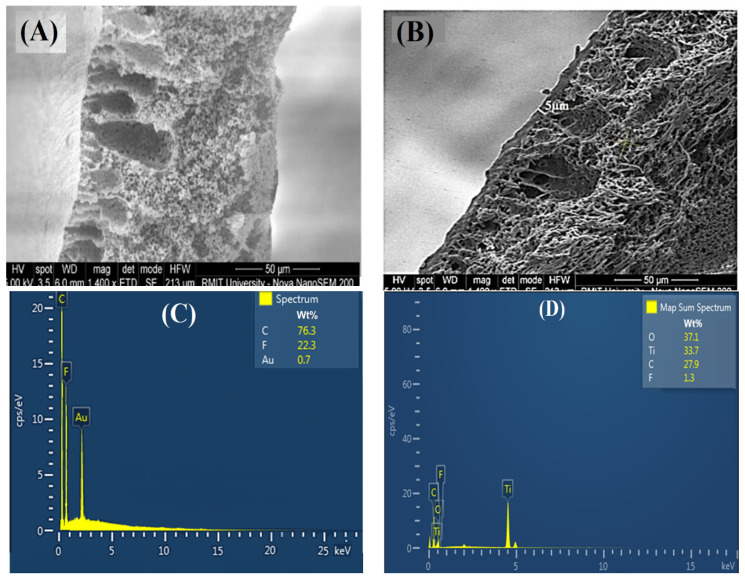
The SEM images of cross-sections of plain and modified PVDF membranes: (**A**) plain PVDF membrane (0 wt.% TiO_2_) and (**B**) TiO_2_/PVA modified PVDF membrane (1 wt.% TiO_2_); and the corresponding EDX images of the top surface of the membrane (**C**) plain PVDF membrane (0 wt.% TiO_2_) and (**D**) TiO_2_/PVA modified PVDF membrane (1 wt.% TiO_2_).

Table 2 indicates that an increase in TiO_2_ loading increases the solution’s viscosity, which leads to the formation of a thick film over the substrate. Figure 4A,B illustrates the cross-sectional images of plain and TFC membranes with 1 wt.% TiO_2_. The TiO_2_ coated membrane has two distinct layers, one with a thin film of TiO_2_/PVA, having a thickness of 5 µm, which did not exist in the plain PVDF membrane. When the solution viscosity increases, the layer’s thickness also increased due to the agglomeration of nanoparticles in the solution. The presence of the TiO_2_ nanoparticles on the membrane surface was confirmed by the Energy-dispersive X-ray spectroscopy (EDX) analysis (Figure 4C,D). The main peak of 0.67 and 0.277 keV represent the fluorine and carbon peak, which are abundant because they signify the PVDF membrane. A peak detected at 4.5 keV belongs to Ti, and 0.52 keV represents oxygen indicating the presence of the TiO_2_ nanoparticles in the composite membrane.

### 3.2. Surface Roughness Analysis of TiO_2_ Composite PVDF Membrane

The surface topography is one of the powerful techniques for mapping the surface morphology, roughness, and adhesive properties of the nanoparticles on the PVDF membrane with and without modification. Figure 3A–F represents the AFM images of the plain and TiO_2_/PVA coated membranes. The average value of root means square (RMS) and the average roughness (Ra) of different membranes are presented in Table 3. It can be observed that the TiO_2_ loading has increased the surface roughness of the modified membrane. AFM images show that the membrane surface possesses peak and valley like structures (the brightest parts in each AFM images represent the peak, whereas the darkest parts represent the valleys). The increasing surface roughness with increasing TiO_2_ loading confirms the adhesion of TiO_2_ nanoparticles on the TFC membrane’s surface. The increase in TiO_2_ loading has also increased the peak and valley, which could help to enhance photocatalysis. When a dye solution passed through the membrane, these valleys can adsorb the dye molecules, which could be successfully degraded by the hydroxyl radicals produced by TiO_2_ under UV irradiation [35].

### 3.3. The Contact Angle of the Membrane

**Figure 5:**
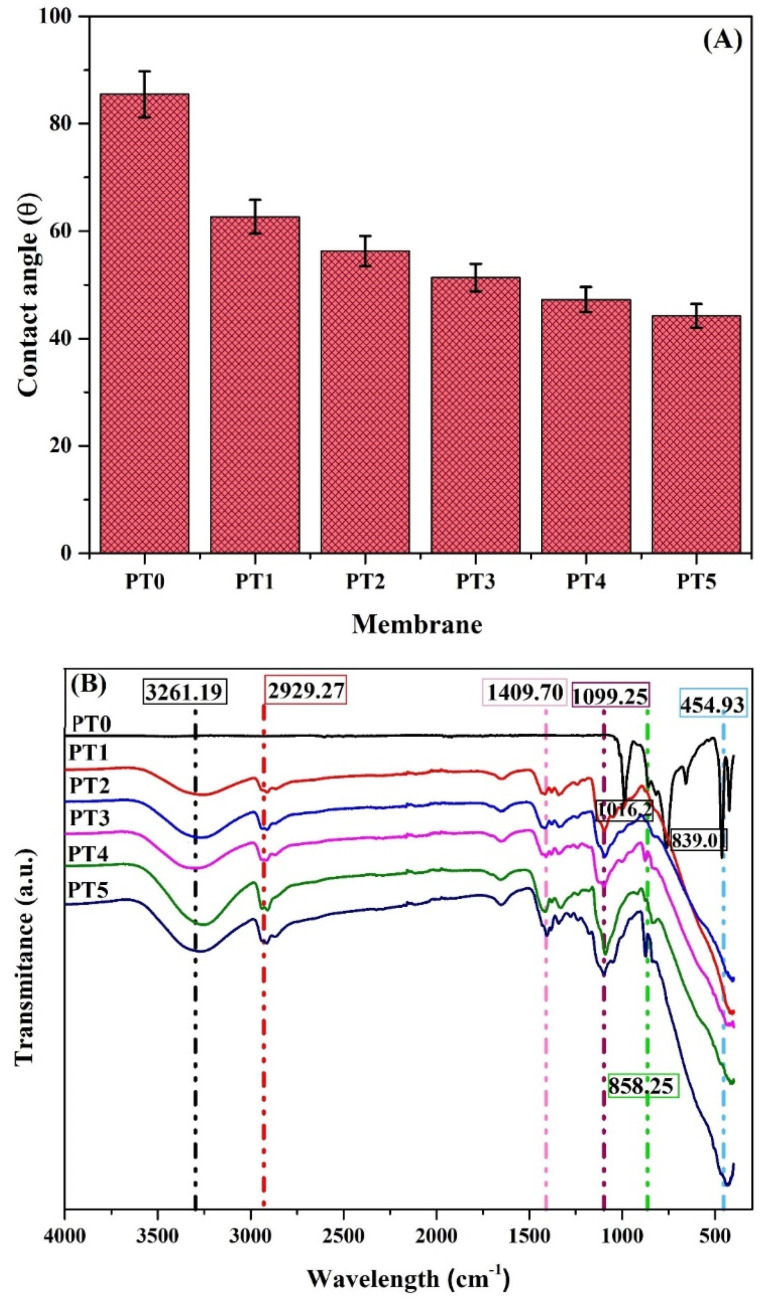

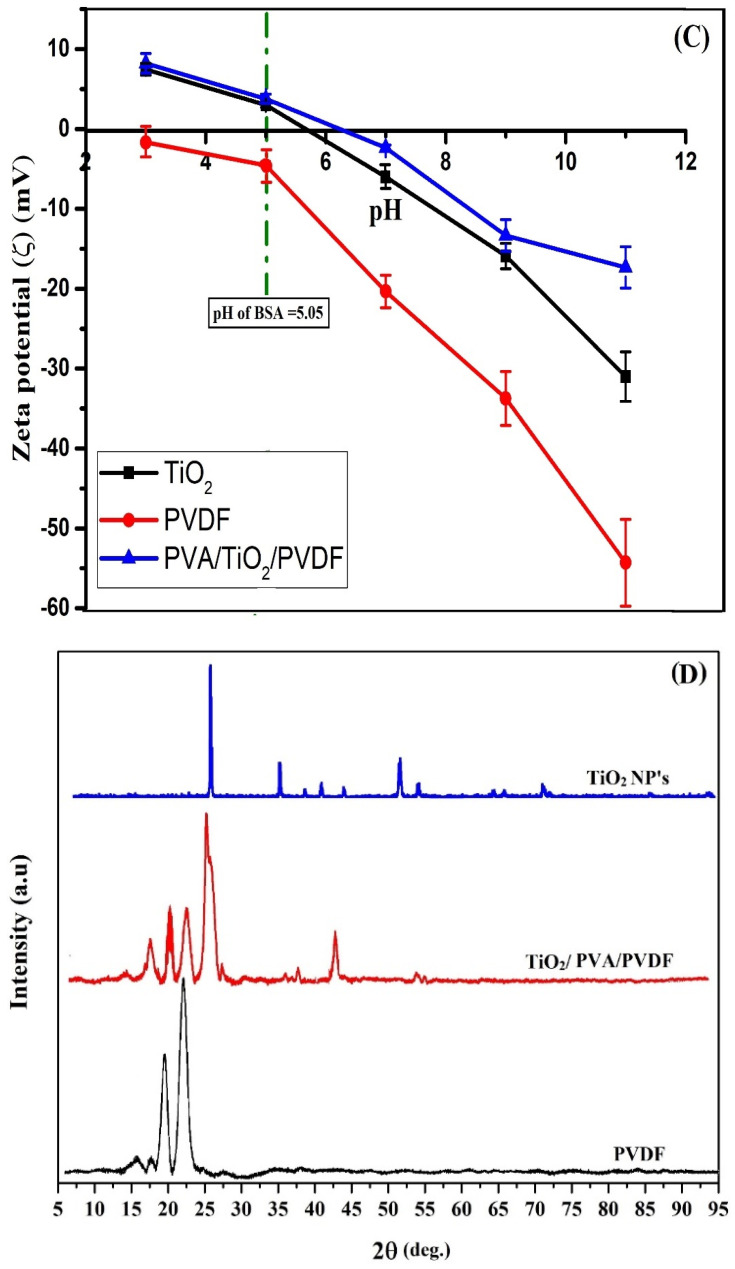
Effect of TiO_2_ loading (**A**) Contact angle of the membranes; (**B**) FTIR spectra of plain and modified PVDF membranes; (**C**) Zeta potential values at different pH for TiO_2_, plain PVDF and 1 wt.% TiO_2_ /PVA modified PVDF membrane; (**D**) XRD pattern of TiO_2_ nanoparticles, plain and modified (for 1 wt.% TiO_2_) PVDF membranes.

The wettability of the samples plays a very decisive role in determining the water permeability of a membrane. Generally, it is considered as the higher the degree of hydrophilicity, the greater the water permeability [36]. The contact angles of the plain and modified PVDF membranes are illustrated in Figure 5A. The plain PVDF membrane contact angle is 87.42° and decreased with the increase in TiO_2_ loading. Membrane with 5 wt.% TiO_2_ shows the lowest contact angle 48ᵒ. The increase in TiO_2_ concentration also implies the increase in PVA on the membrane surface, which creates the hydroxyl groups on the surface [31]. The increase in the hydroxyl group enhances the hydrophilicity of the membrane. Mänttäri et al. [37] found that a membrane with a lower contact angle exhibit a significant change in zeta potential with an increase in pH.

### 3.4. FTIR Analysis

Fourier-transform infrared (FTIR) spectroscopy was used to study the change in the functional group on the membrane surface after the modification by TiO_2_/PVA thin film coating (with increasing TiO_2_ loading). The measurements were taken from 4000 to 400 cm^−1^**,** and the functional groups that participated in the structure were determined from the peaks of the graphs of transmission against the wavelength. In Figure 5B, the absorption band at 1140–1280 cm^−1^ and 1411–1419 cm^−1^ is characteristic stretching of CF_2_ and CH_2_ group, respectively, found in the PVDF membrane [38]. The PVDF membrane also showed peaks at 873.88, 832.88, and 476.86 cm^−1^ which have been associated with the C-C-C bond. A distinct peak at 2929.27, 1409.70, and 1099.25 cm^−1^ resulted in the stretching of ‑CH_2_, C=O, and C-O, respectively, which are the characteristics peak of PVA [39]. A broad peak in the TiO_2_/PVA composite membrane at 2900–3500 cm^−1^ represents the Ti-OH group, which shows the presence of -OH hydrophilic group on the surface and indicates the presence of TiO_2_ nanoparticles [40]. The increase in TiO_2_ loading enhanced the intensity of the peak at 3258.95 cm^−1^. The increase in peak intensity of -OH band represents the increase in TiO_2_ nanoparticles on the surface of all the membranes (PT1 to PT5 ).

### 3.5. Surface Charge of the Membrane

The surface charge has a significant effect on the removal efficiency and membrane fouling. The surface charge can be characterized in terms of zeta potential, as illustrated in Figure 5C. Zeta potential (ζ) changed with pH of the solution due to protonation and deprotonation of the membrane’s functional group. Its values, either positive or negative, have a substantial effect on stabilizing the particles in the suspension. This is attributed to the electrostatic repulsion between particles with the same electric charge that causes the particles’ segregation [41]. The PVDF membrane is negatively charged over the entire pH range due to the C-F group’s electronegative charge [42], whereas TiO_2_ particles show positive zeta potential in acidic conditions and start to fall off with the increase in pH. The zeta potential of TiO_2_/PVA coated PVDF membrane is positive in acidic conditions and becomes negative in alkaline conditions. The acquired positive charge in acidic conditions indicates the presence of TiO_2_ on the PVDF membrane. The decrease in the zeta potential value with the increase in pH is due to the surface adsorption of OH^−^ and Cl^−^ anions from the solution.

### 3.6. Crystalline Structure of the Synthesized Membranes

Crystallinity is one of the important parameters which affects the chemical and mechanical properties of a polymer. The XRD diffraction patterns of TiO_2_ nanoparticles, plain PVDF membrane, and TiO_2_/PVA coated PVDF membrane containing 1 wt.% of TiO_2_ nanoparticles are shown in Figure 5D. TiO_2_ is a mixture of two different forms (75% anatase and 25% rutile); therefore, the TiO_2_ pattern has three dominating peaks at 2θ = 25.25°, 37.12°, and 48.05° [43,44]. The diffraction peaks at 2θ = 20.8° and 18.56^°^ are the characteristics of α-phase PVDF polymer, which were also observed in the TiO_2_/PVA modified. It consists of characteristic peaks at 19.5° and 38.6°, which are related to the semi-crystalline nature of PVA [18,30,45]. The diffraction peaks at 25.2 and 45.89 indicate the anatase structure of TiO_2_ [46]. It indicates that nano-TiO_2_ particles did not change the crystal structure of TiO_2_ on the surface after modification.

### 3.7. Performance and Photocatalytic Activity of the Membrane

#### 3.7.1. Flux and Removal of Dyes by Composite Membranes

**Figure 6:**
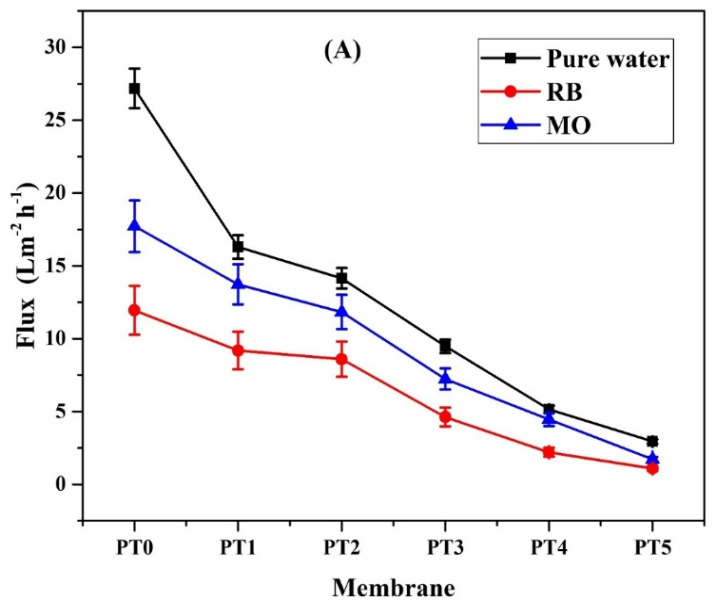

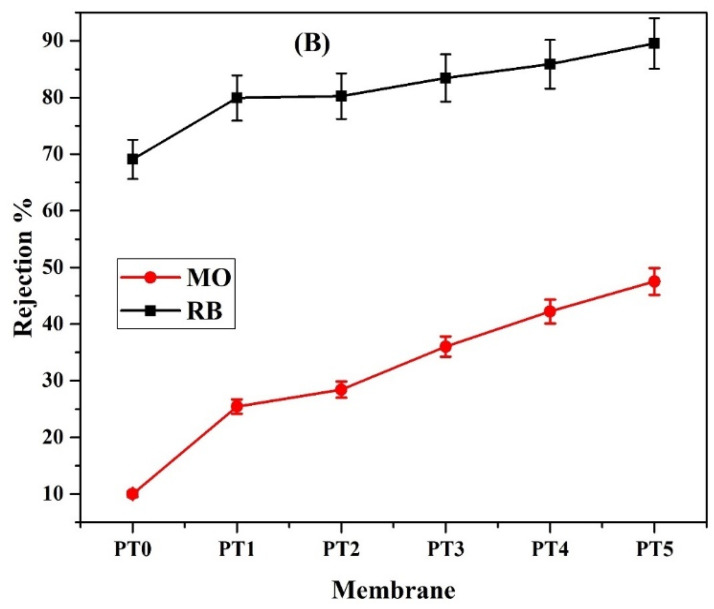

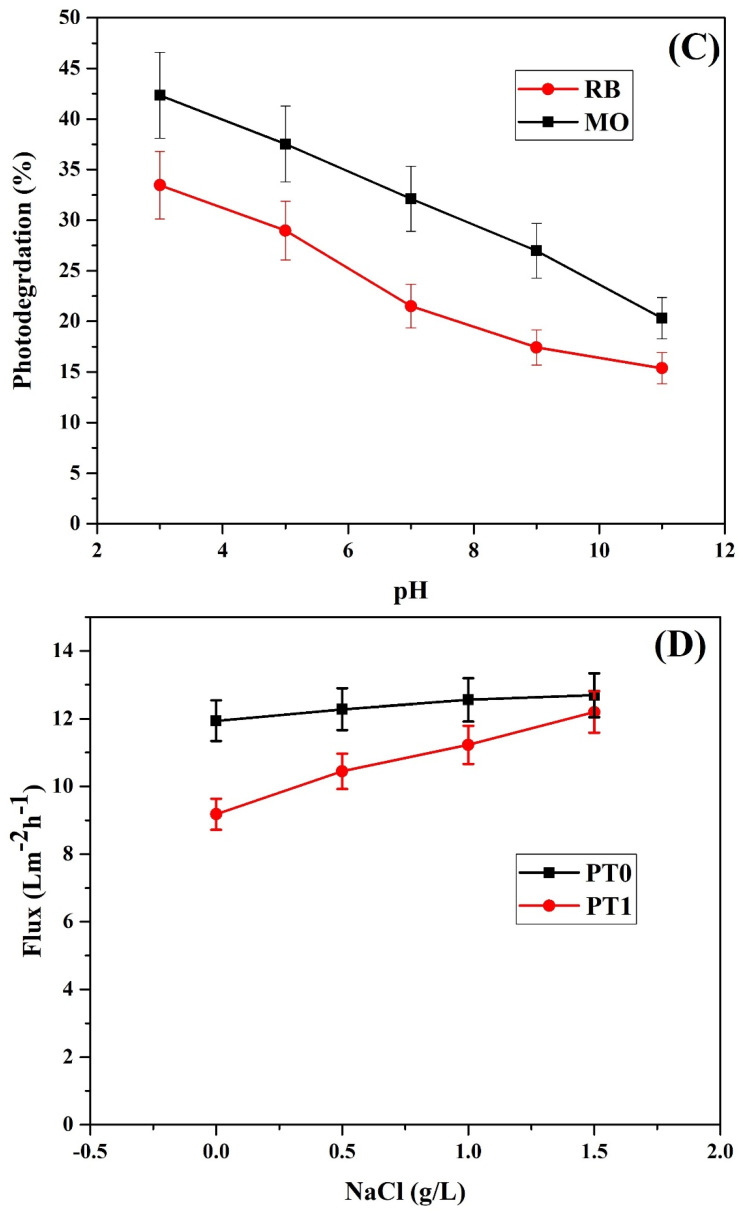

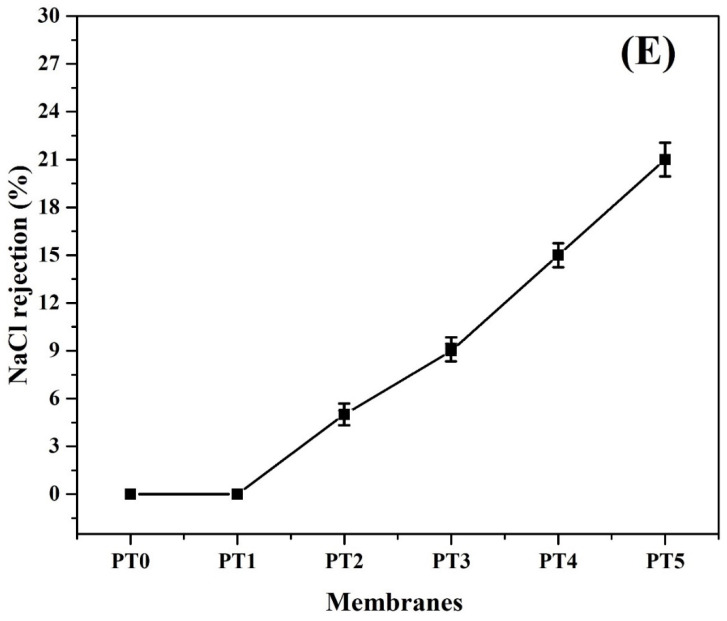
(**A**) Average flux for modified and unmodified PVDF membranes at different TiO_2_ loading (0 to 5 wt.%) at an operating pressure of 3 bar for pure water and the dye solutions (RB and MO) (Dye concentration = 50 mg/L); (**B**) Rejection of dyes by TiO_2_/PVA modified and unmodified PVDF membrane during filtration (Dye concentration = 50 mg/L and experimental duration = 60 min);(**C**) Effect of solution pH on photodegradation of dyes (MO and RB) at 50 mg/L of dye concentrations under UV irradiation (Time =180 min); (**D**) Effect of NaCl concentration on RB (50 mg/L) treatment (P = 0.3 MPa); (**E**) NaCl rejection at different TiO_2_ loading.

The variation in pure water flux of membranes coated with TiO_2_ (0, 1, 1.5, 2, 3, 5 wt.%) nanoparticles was evaluated at 3 bar and presented in Figure 6A. The incorporation of TiO_2_ on the membrane surface results in blocking the surface pores and causing the reduction in the permeate flux [47]. SEM images also show that an increase in the loading of TiO_2_ nanoparticles improves the dispersions with the formation of a thin film of TiO_2_/PVA over the PVDF membrane’s surface, which results in the decrease in pore size. A gradual reduction in pure water flux was observed as the TiO_2_ loading was increased in the membranes from PT1 to PT5. The pure water flux for all the membranes is comparatively higher than the permeate flux obtained for dye solutions due to the accumulation of dye molecules on the membrane surface, causing membrane fouling and therefore reducing the membrane flux. Comparing to RB dye, the permeate flux obtained for MO dye solution was slightly higher. Lower molecular weight dye (MO) can easily pass through the membrane compared to the higher molecular weight dye (RB), which can accumulate on the membrane surface and yielding lower permeate flux.

Rejection of different dyes with membranes coated with different TiO_2_ concentrations (0 to 5 wt.%) was studied at a temperature of 27 ± 3 °C at an operating pressure of 3 bar. The concentrations were 50 mg/L for both dyes, and the filtration was continued for 60 min. Figure 6B indicates that the modified membrane with TiO_2_/PVA improved dye rejection compared to the plain PVDF membrane. The rejection values better for RB dye due to its higher molecular weight, whereas MO dye showed low rejection values. It implies that the dye with higher molecular weight can be adsorbed easily onto the membrane surface [48]. Almost complete removal of dye was obtained in the case of PT5 membrane but with the lowest permeate flux of 0.95 Lm^−2^h^−1^.

#### 3.7.2. Effect of Solution pH on TFC Membrane

pH is considered an important parameter in determining the removal performance and efficiency of the photocatalytic process. The influence of solution pH in the removal of RB and MO was studied by changing the solution pH from 3 to 11, and the results are shown in Figure 6C. Tests were conducted with an initial concentration of 50 mg/L of RB and MO, at a minimum TiO_2_ loading (1 g/L) under UV irradiation. According to Figure 6C, RB and MO degradation is higher in acidic conditions and decreased in alkaline conditions. In the feed solution, the dye molecule state is changed due to pH, which influenced the rate of photodegradation [31]. In Figure 5C, at pH less than 5.9, the surfaces of TiO_2_ nanoparticles are positively charged, whereas changed to negative charge at pH higher than 5.9. Thus, pH lower than that corresponding to the point of zero charge supports the adsorption of dye (RB and MO) molecules on the surface of the membrane, which improves the degradation of dyes under acidic and neutral condition.

Furthermore, Nakabayashi et al. [49] explained that at lower pH value, TiO_2_ nanoparticles could produce higher concentrations of hydroxyl ions, which subsequently cause an increased photocatalytic degradation. Additionally, the increase in pH value increases the coulomb repulsion between the negative surface charge of the TFC membrane and the OH radicals in the photocatalytic oxidation leading to a decrease in photodegradation [50].

#### 3.7.3. Effect of Salt on TFC Membrane

Textile mills commonly use salts for the dying process and most of which are discharged in the effluent and can have adverse effects on the dye removal process. Therefore, the effect of NaCl salt with RB dye solution (50 mg/L) was studied with and without PVA/TiO_2_ coated PVDF membrane (Figure 6D). An increase in the salt concentration increased the permeate flux rate for the TiO_2_/PVA coated membrane compared to that for the plain PVDF membrane. This is due to the Donnan effect between the surface groups (hydroxyl group) of the TiO_2_/PVA coated membrane and the ions with the same charge weakened with the increase in salt concentration and increased flux [10]. The increasing salt concentration could cause thinning of PVA/TiO_2_ layer on the membrane surface due to charge interactions [31].

The effect of TiO_2_ loading on the removal of salt is represented in Figure 6E. The NaCl rejection increased from 0% for PT0 to 21% for PT5. The deposition of TiO_2_/PVA film on the PVDF membrane’s surface might increase the film thickness, which lowers the flux but enhances the salt rejection. The obtained results indicate that the increasing TiO_2_ loading in the TFC membrane improves permeability and ion rejection.

#### 3.7.4. Fouling Study of PVA/TiO_2_ TFC PVDF Membrane

**Figure 7:**
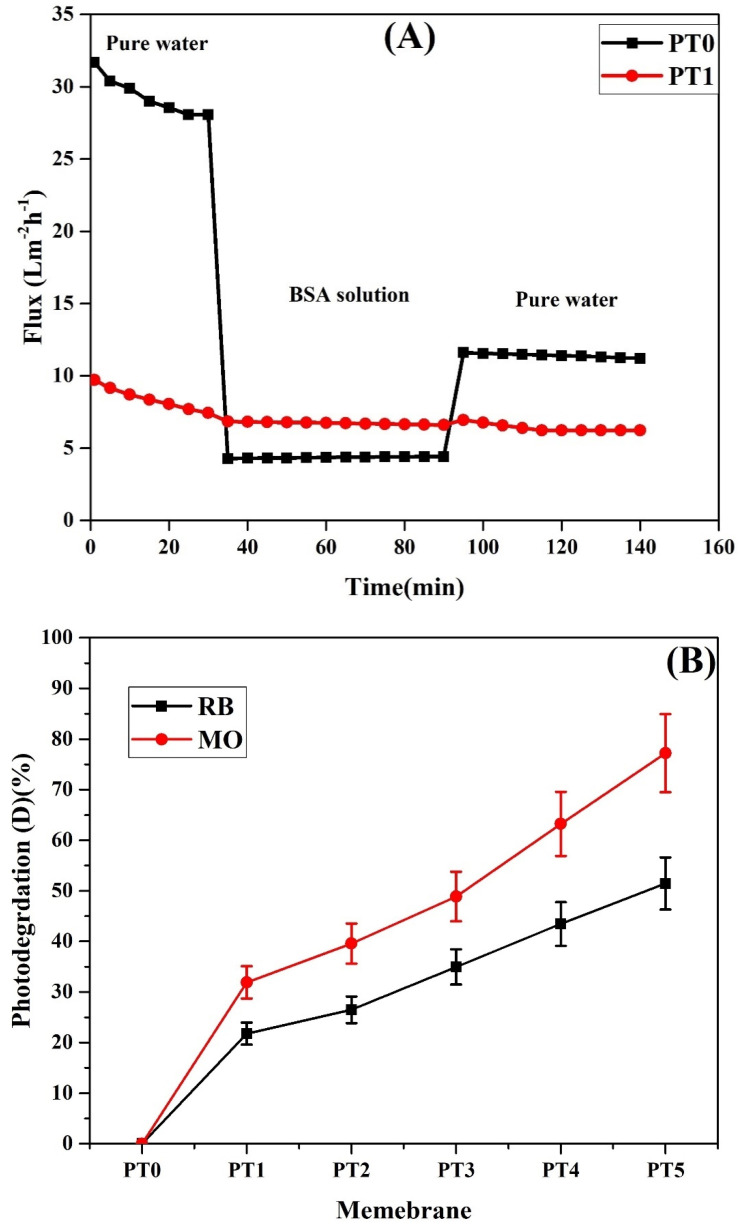
(**A**) Bovine serum albumin (BSA) removal using plain and modified membranes (PT0 and PT1) (BSA concentration = 1 g/L, operating pressure = 3 bar); (**B**) Photocatalytic degradation of dyes (reactive blue and methyl orange) at a dye concentration of 50 mg/L under UV irradiation for 150 min.

The fouling behavior of the TiO_2_/PVA PVDF membrane was carried out using the BSA solution (1 g/L) for 60 min at a pressure of 3 bar. The data from Figure 7A indicates that the initial flux of plain PVDF membrane (PT0) was higher, but the start to decline during the BSA solution’s filtration. Whereas the TiO_2_ coated membrane shows similar flux before and during the BSA rejection. The results reveal that the antifouling properties of TiO_2_/PVA coated membrane has improved considerably compared to that of the plain PVDF membrane. Moreover, the increase in TiO_2_ loading enhanced the fouling mitigation properties of the membrane. Polymer surfaces are more reactive to protein molecules than TiO_2_ molecules; therefore, incorporating TiO_2_ in PVDF membrane prevents the adsorption of protein on the membrane surface [51]. The presence of TiO_2_ nanoparticles helps to wash away the protein molecules from the composite membranes compared to the plain PVDF membrane. Wang et al. [52] have observed that the accumulation of TiO_2_ nanoparticles boosted the electron donor mono-polarity of the TiO_2_/PVA composite membranes and enhanced the repulsive interaction energy between foulants and membrane surfaces to improve the antifouling ability.

Further, the zeta potential of the plain and modified PVDF membrane containing 1 wt.% TiO_2_ at pH 5 were −4.56 and 3.78 mV, respectively. The pH of the BSA solution was also around 5, and thus both the modified PVDF membrane and BSA might have the same surface charge (positive), leading to the resistance by the membrane to adsorb BSA.

The pure water flux recovery for the PT1 membrane was 76.2% after filtering BSA through it, whereas for the plain PVDF membrane, the pure water flux recovery was about 38.9%. The results also indicate that the irreversible fouling factors for the modified (PT1) and unmodified (PT0) PVDF membranes were 25 and 61%, respectively.

#### 3.7.5. Photocatalytic Performance and Degradation Kinetics at Different TiO_2_ Concentration

Figure 7B illustrates the effect of TiO_2_ loading on the reactive blue and methyl orange dye’s photodegradation at a concentration of 50 mg/L. The photocatalytic performance of the membranes was evaluated by measuring the change in the concentration of dyes for a duration of 180 min (for the initial 30 min, the membrane was soaked in the dye solution and kept in the dark, and for the rest of the time, the membrane was soaked in the dye solution and exposed to UV irradiation). The results indicated that the adsorption of the dyes was less than 5% in the dark.

**Figure 8:**
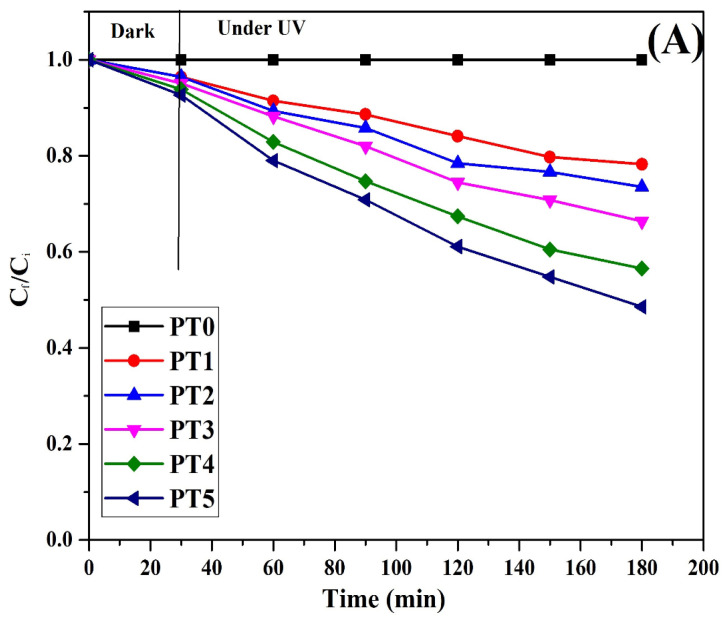

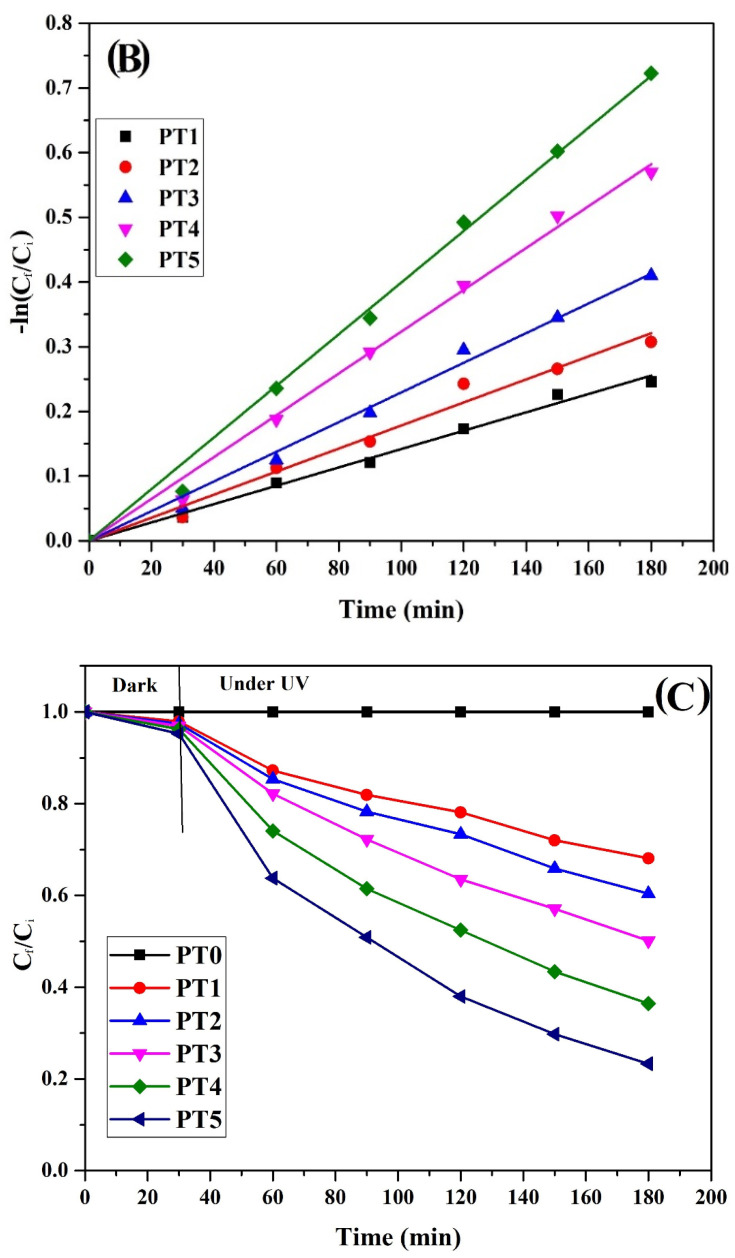

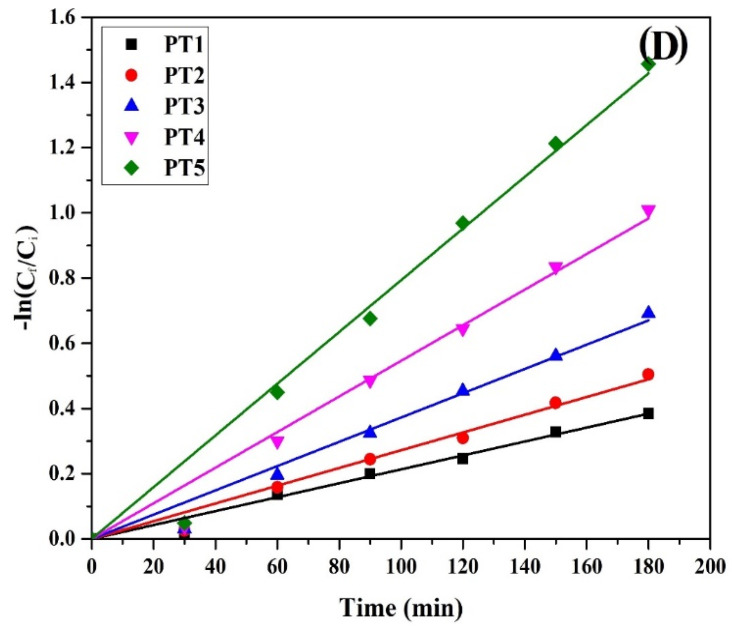
Temporal variation of normalized dye concentration in the permeate obtained from the plain and the modified PVDF membranes: (**A**) RB (**C**) MO; Natural log plots of normalized dye concentration in the permeate obtained from the plain and the modified PVDF membranes against time to compute rate constants: (**B**) RB (**D**) MO.

The temporal change in the concentration of RB and MO in the presence of different membranes with and without TiO_2_ are illustrated in Figure 8A,C. Results indicate that the membranes with TiO_2_ nanoparticles show significant photocatalytic activity compared to that of the plain PVDF membrane. The plain PVDF membrane does not show any removal under UV irradiation, whereas the dye degradation rate increases for the TiO_2_/PVA coated membrane. The increase in TiO_2_ loading (0 to 5 wt.%) enhanced the photo degradability of the dyes due to the generation of the hydroxyl group. The higher the concentration of TiO_2_, the higher the hydroxyl radicals produced, which enhanced the degradation rate. The maximum degradation of 77.2% for MO and 51.5% for RB were achieved under UV irradiation for the PT5 membrane. This was due to the generation of active radical species such as positive holes (h^+^), hydroxyl radicals (OH^•^), and superoxide radicals (O_2_^•^) on the surface of the modified membranes due to the incorporation of TiO_2_ nanoparticles. Those radicals attack the dye molecules, causing their degradation into intermediates through various reaction followed by the complete mineralization into less toxic CO_2_ and H_2_O.

To quantify the photocatalytic effects on the degradation of dyes at different TiO_2_ loading, the Langmuir-Hinshelwood (L-H) kinetic model was used [53].

(5) $$-\frac{\mathrm{dC}}{\mathrm{dt}}\;=\frac{\mathrm{kKC}}{1+\mathrm{KC}}$$

After integrating the above equation, we can obtain the following relationship:

(6) $$\ln\left(\frac{C_{i}}{C_{f}}\right)+K\left(C_{i}-C_{f}\right)=\mathrm{kKt}$$

where C_i_, C_f_, are the initial and final concentrations of the dye at time 0 and t, respectively; k and K are the reaction rate constant and adsorption equilibrium constant, respectively. C is the concentration at a given time, t. The order of Equation (5) will be zero when C_f_ is relatively high, and KC is ≫1. When the concentration of dye in the solution decreases, then KC will become ≪1. Thus, the KC in the denominator of Equation (5) will be negligible, and the reaction will be the first order. Overall, dyes’ degradation rate was increased with the increase in the concentration of TiO_2_ nanoparticles over the surface of the membrane. Thus, assuming the first-order decay of dyes, the following equation can be used:

(7) $$-\frac{\mathrm{dC}}{\mathrm{dt}}=\mathrm{kKC}=k^{\prime }C$$

Integrating the Equation (7) will yield Equation (8):

(8) $$-\ln\frac{C_{f}}{C_{i}}\;=k\prime t$$

The linear regressions obtained from the plots −ln (C_f_/C_i_) v/s time is shown in Figure 8B,D, and the values of the rate constants are listed in Table 4. The half-reaction time, t_1/2_ required for the concentration to drop one-half of its initial concentration can be given by Equation (9):

(9) $${t\;}_{1/2}=\;\frac{\ln\left(2\right)}{k^{\prime }}$$

The results indicate that the increase in TiO_2_ concentration in the film enhanced the reaction rate and decreased the half-reaction time. The shortest half reaction time corresponds to the high rate of degradation.

#### 3.7.6. The Mechanisms of Photodegradation of Dyes by TFC Membrane

**Figure 9:**
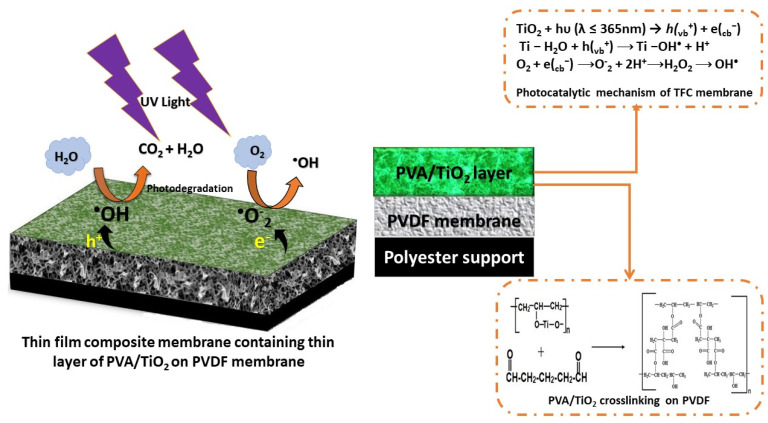
Photocatalytic mechanism of TFC membrane under UV irradiation.

The detailed mechanism of the degradation and the antifouling process of the TFC PVDF membrane is explained below (Figure 9). The mechanisms of photodegradation are photoexcitation, migration, oxidation-reduction reaction, and charge separation [45]. When the TiO_2_ coated surface is exposed to UV irradiation (hυ) with the energy equal or higher than the bandgap energy, electrons are excited from valence band (VB) to conduction band (CB), generating holes (*h*_vb_^+^) at VB and electrons (e_cb_^−^) at CB. Holes and electrons react with species adsorbed on the surface of the TiO_2_ catalyst. Valence band holes react with water (H_2_O/OH^−^) to generate hydroxyl radicals (HO^•^), while electrons react with adsorbed molecular oxygen (O_2_) and reducing it to superoxide radical anion, which, in turn, reacts with protons to form peroxide radicals [54,55] The relevant reaction that occurs on the surface of the TFC PVDF membrane for the photodegradation of the RB and MO can be described as:

TiO_2_ + hυ (λ ≤ 365 nm) → *h(*_vb_^+^) + e(_cb_^−^) (10)

The electron from CB is readily surrounded by the O_2_ to generate superoxide radicals (O_2_^•⁻^) on the surface of TiO_2_

e(_cb_^−^) + O_2_ → (O_2_^•⁻^) (11)

The generated superoxide radicals (O_2_^•⁻^) could react with the water molecules (H_2_O) to produce hydroxyl radicals (OH^•^) and hydroperoxyl radicals (HO_2_^•^) which are the strong oxidizing agents to degrade the organic molecules.

Whereas the holes produced on VB surrounded by the hydroxyl group (H_2_O) to generate hydroxyl radicals (OH^•^) on the surface of TiO_2_.

*h*(_vb_^+^) + OH^−^ → OH^•^ (12)

In an aqueous medium, VB holes can react with the surface adsorbed water molecules to form hydroxyl species.

Ti − H_2_O + h(_vb_^+^) → Ti − OH^•^ + H^+^ (13)

Therefore, when the dye (RB and MO) molecules react with the hydroxyl radicals, they oxidized into CO_2_ and H_2_O as follows;

C_14_H_14_N_3_NaO_3_S (MO) or C_22_H_16_N_2_Na_2_O_11_S_3_ (RB) + OH^•^ +O_2_ ⟶ Product (CO_2_) and (H_2_O) + degradation product (14)

Further, our main focus is to modify the PVDF membranes and understand morphological behavior. The absorption of the dyes was studied under dark condition and with UV irradiation. The change in concentration was calculated by observing the change in absorption at a maximum wavelength (MO = 464 and RB = 590). As per Deng et al. [56], many organic dyes such as erythrosine, eosin Y, rhodamine B, and rose bengal have been utilized to sensitize catalysts for H_2_ production. Despite MO and RB are organic dyes, they are not utilized for catalyst sensitization. Further studies are warranted in this area.

### 3.8. Stability of TiO_2_ Nanoparticles Coated onto the Membrane Surface during Photocatalysis

**Figure 10:**
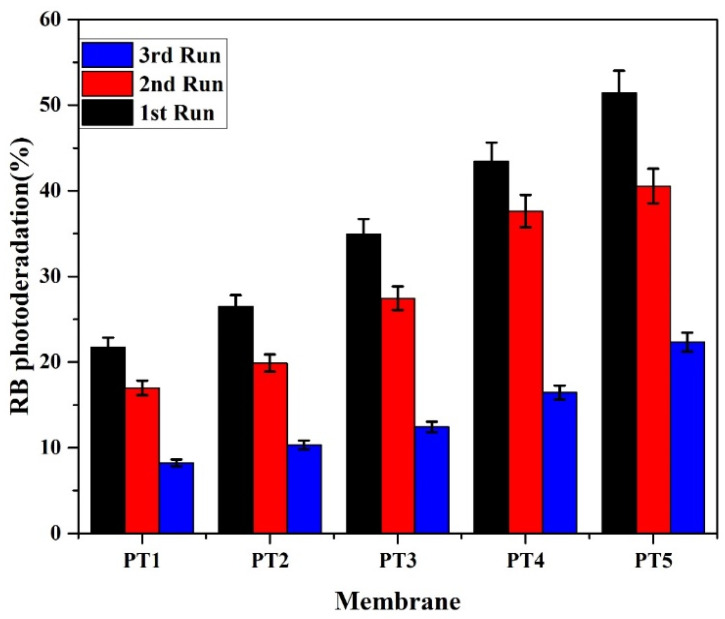
Degradation performance (stability) of TiO_2_ nanoparticles while degrading reactive blue dye under UV irradiation during repeated runs (Dye concentration = 50 mg/L, 150 min. of UV irradiation per run).

The stability of the TiO_2_ nanoparticles over the surface of the membrane has an essential role in the membrane’s performance. The long-term stability of the modified membrane has been tested by degrading RB repeatedly three times. In order to understand the stability of TiO_2_ nanoparticles on the TiO_2_/PVA composite PVDF membrane, the membrane was reused for the photodegradation of the RB dye. TiO_2_/PVA composite membranes were successively used three times for the dye degradation; after each use, the membranes were washed with milli-Q water and dried at room temperature. A fresh batch of dye solution (50 mg/L) was prepared for the next cycle, and the experiment was performed under the same operating conditions. Figure 10 shows that the photodegradation rate decreased in the second and third runs, compared to that in the first run. The membrane with higher TiO_2_ (PT5) showed a change in photodegradation of RB dye (~24 %) after the third cycle. Therefore, we conclude that the TiO_2_/PVA thin film coating is suitable for the degradation of dyes under UV irradiation. The loss in photocatalytic efficiency is mainly due to the loss of TiO_2_ nanoparticles during the washing of membranes [57].

## 4. Conclusions

In this research, the photocatalytic activity of the TFC PVDF membrane was studied to treat the aqueous solution of reactive blue and methyl orange dyes. Membranes synthesized by dip coating at different TiO_2_ loading were characterized using spectroscopic techniques. Based on the SEM, FTIR, and XRD measurements, the presence of TiO_2_ nanoparticles on the surface of the membrane was confirmed. PVA helps to obtain better efficacy of TiO_2_ nanoparticles over the PVDF membrane’s surface. Glutaraldehyde is a cross-linker for the PVA polymer chains to enhance the thin film mechanical and chemical stability. The TFC membrane showed better removal of dyes, enhanced antifouling ability, and hydrophilicity compared to the plain PVDF membrane. The enhancements of surface negative charge and hydrophilicity made the TFC membrane more resistant to the deposition of dyes. The increasing concentration of TiO_2_ nanoparticles in thin-film coating improved the photocatalytic activity and the rate of degradation of dyes. It was found that the degradation of RB and MO fitted the first-order kinetics.

As the TiO_2_ loading increases in the thin film, nanoparticles start to aggregate, reducing the porosity and the permeate flux of the membrane. The stability of nanoparticles remained for three experimental cycles with a slight decrease in photodegradation capacity. The attractive results provide valuable insights into the design and fabrication of high-performance polymeric TFC membrane and demonstrate a promising approach for the efficient removal of dyes in an aqueous solution.

## Figures and Tables

**Table 1:** Physio-chemical properties of the dyes used in this study.

Dye	Abbreviation	Chemical Formula	Chemical structure	Molecular Weight (g/mol)	Type	Wavelength at Which the Maximum Absorbance Occurred, λ_max_ (nm)
Methyl orange	MO	C_14_H_14_N_3_NaO_3_S	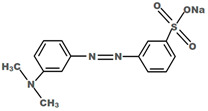	626.50	Azo dye	464
Reactive blue	RB	C_22_H_16_N_2_Na_2_O_11_S_3_	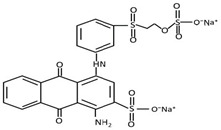	327.33	Anthraquinone dye	590

**Table 2:** Composition of TFC membranes and the viscosity of coating solutions (TiO_2_/PVA).

Membrane (Abbreviation)	Modified Solution Composition (TiO_2_: PVA) (wt.%/wt.%)	Viscosity (Pa. s)
Membrane 1 (PT0)	Plain PVDF	
Membrane 2 (PT1)	1:3	0.7 ± 0.1
Membrane 3 (PT2)	1.5:3	1.3 ±0.2
Membrane 4 (PT3)	2:3	1.5 ±0.1
Membrane 5 (PT4)	3:3	2.4 ±0.2
Membrane 6 (PT5)	5:3	3.7 ±0.3
(in all TFC membranes, PVDF = 16 wt.% and DMAc = 84 wt.%)		

**Table 3:** Surface roughness parameters at different TiO_2_ loading on the PVDF membranes obtained from AFM images (Figure 3).

Membranes	Root Mean Square Roughness (RMS) (nm)	Mean Roughness (Sa) (nm)
PT0	1.07	0.9
PT1	4.46	3.5
PT2	20.33	13.4
PT3	37.45	24.40
PT4	80.40	45.43
PT5	118.20	102.5

**Table 4:** The apparent first-order rate constant (k′) and half-life time of MO and RB degradation by plain and modified PVDF membranes.

Membrane	MO			RB		
	Rate Constant k′ (min^−1^)	R^2^	t_½_ (min)	Rate Constant k′ (min^−1^)	R^2^	t_½_ (min)
PT1	0.0021	0.993	330	0.0014	0.997	495
PT2	0.0027	0.992	256.66	0.0017	0.994	407.64
PT3	0.0037	0.991	187.29	0.0022	0.997	315
PT4	0.0054	0.991	128.33	0.0032	0.998	216.56
PT5	0.0079	0.991	87.72	0.0039	0.998	177.69

## Data Availability

Data is contained within the article.
